# Interaction analysis of statistically enriched mutations identified in Cameroon recombinant subtype CRF02_AG that can influence the development of Dolutegravir drug resistance mutations

**DOI:** 10.1186/s12879-021-06059-x

**Published:** 2021-04-23

**Authors:** Sello Given Mikasi, Darren Isaacs, Rumbidzai Chitongo, George Mondide Ikomey, Graeme Brendon Jacobs, Ruben Cloete

**Affiliations:** 1 Division of Medical Virology, Department of Pathology, Faculty of Medicine and Health Sciences, Stellenbosch University, Tygerberg, South Africa; 2 South African Medical Research Council Bioinformatics Unit, South African National Bioinformatics Institute, University of the Western Cape, Robert Sobukwe Rd, Bellville, P.O. Box X17, Cape Town, 7535 South Africa; 3 Centre for the Study and Control of Communicable Diseases, Faculty of Medicine and Biomedical Sciences, University of Yaoundé I, Yaoundé, Cameroon

**Keywords:** Cameroon, Integrase, CRF02_AG, Molecular modelling, Interaction analysis

## Abstract

**Background:**

The Integrase (IN) strand transfer inhibitor (INSTI), Dolutegravir (DTG), has been given the green light to form part of first-line combination antiretroviral therapy (cART) by the World Health Organization (WHO). DTG containing regimens have shown a high genetic barrier against HIV-1 isolates carrying specific resistance mutations when compared with other class of regimens.

**Methods:**

We evaluated the HIV-1 CRF02_AG IN gene sequences from Cameroon for the presence of resistance-associated mutations (RAMs) against INSTIs and naturally occurring polymorphisms (NOPs), using study sequences (*n* = 20) and (*n* = 287) sequences data derived from HIV Los Alamos National Laboratory database. The possible impact of NOPs on protein structure caused by HIV-1 CRF02_AG variations was addressed within the context of a 3D model of the HIV-1 IN complex and interaction analysis was performed using PyMol to validate DTG binding to the Wild type and seven mutant structures.

**Results:**

We observed 12.8% (37/287) sequences to contain RAMs, with only 1.0% (3/287) of the sequences having major INSTI RAMs: T66A, Q148H, R263K and N155H. Of these,11.8% (34/287) of the sequences contained five different IN accessory mutations; namely Q95K, T97A, G149A, E157Q and D232N. NOPs occurred at a frequency of 66% on the central core domain (CCD) position, 44% on the C-terminal domain (CTD) position and 35% of the N-terminal domain (NTD) position. The interaction analysis revealed that DTG bound to DNA, 2MG ions and DDE motif residues for T66A, T97A, Q148H, N155H and R263K comparable to the WT structure. Except for accessory mutant structure E157Q, only one MG contact was made with DTG, while DTG had no MG ion contacts and no DDE motif residue contacts for structure D232N.

**Conclusions:**

Our analysis indicated that all RAM’s that resulted in a change in the number of interactions with encompassing residues does not affect DTG binding, while accessory mutations E157Q and D232N could affect DTG binding leading to possible DTG resistance. However, further experimental validation is required to validate the in silico findings of our study.

## Background

Sub-Saharan Africa (SSA) remains one of the regions highly burdened by HIV infection at 70% of the global epidemic. SSA has a particularly high HIV-1 genetic diversity and it is documented that diverse subtypes may affect the clinical treatment outcome in patient management [1]. The HIV-1 CRF02_AG strain continues to be the predominant subtype causing majority of infections in Cameroon, while other strains, including groups N, O and P, account for a minor proportion of infections [2–4]. Furthermore, different mutational pathways account for subtype specific differences in drug resistance [5–7]. Additionally, other studies have also reported that natural occurring polymorphisms (NOP) which are associated with the occurrence of resistance to Integrase (IN) strand-transfer inhibitors (INSTIs) and IN activity, are subtype-dependent [6–8]. These subtype-specific polymorphic mutations in the IN gene have been shown to affect IN DNA binding affinity, in the presence of resistance-associated mutations (RAMs) [6–8]. Computational modelling of RAMs against INSTIs, across different HIV-1 subtypes compared to subtype B, showed that the presence of M50I in subtypes A and C, L74I in subtypes A and CRF02_AG, G163R in CRF01_AE, and V165I in subtypes F and CRF01_AE are associated with a lower genetic barrier to resistance in non-B clades [9]. Cameroon has seen a substantial reduction of HIV infection, since the introduction of combination antiretroviral therapy (cART), especially with the rolling-out of programmes like prevention of mother-to-child transmission (PMTCT) and the implementation 90–90-90 strategy to end the AIDS pandemic by 2030 [10]. The ability of the HIV-1 virus to mutate during therapy, can lead to the emergence of HIV-1 drug resistance and this necessitates the need for more effective cART regimens with higher genetic barriers [1]. In Cameroon, the HIV-1 drug resistance rates among cART-initiators stand at approximately, 10% of the Cameroon infected population [11, 12].

The United States of America (USA) Food and Drug Administration (FDA) has approved four HIV-1 INSTIs, including raltegravir (RAL), elvitegravir (EVG), dolutegravir (DTG) and bictegravir (BIC) [13]. However, the high cost of INSTIs, has resulted in restricted access to this class of drugs in resource-limited countries [14]. Despite the cost, the World Health Organization (WHO) has given the green light to include DTG to an alternative 1st-line regimen [14, 15]. The strand transfer reaction catalyzed by HIV-1-expressed IN enzyme is blocked by the activity of INSTIs which bind to the catalytic site in the catalytic core domain (CCD) of the IN protein [16, 17]. Mutations that confer resistance to INSTIs (for example G140S, Q148H and N155H) have been structurally mapped in close proximity to the IN catalytic active site [18, 19]. Primary resistance to INSTIs, along with residues associated with catalytic activity among different subtypes are highly conserved. HIV-1 sequence and structure-based analyses have shown that polymorphic residues can cause subtype-specific effects, which significantly affect the native protein structure, function and activity important for drug-mediated inhibition of enzyme activity [9]. There is limited information available on the IN structure of CRF02_AG [9, 20] and even less on the effect of mutations on the protein structure. There is therefore a need to continue monitoring patients to identify additional RAMs and polymorphic mutations that might affect the genetic barrier to the development of RAMs against INSTI [9]. The goals of this study was to analyse the Cameroonian CRF02_AG IN gene sequences obtained from the Los Alamos National Laboratory (http://www.hiv.lanl.gov/) HIV-1 database to assess the occurrence of mutations and natural occurring polymorphisms (NOPs). NOPs are categorized under secondary mutations which on their own play a limited role in resistance [14]. However, their pre-existence might favour a more rapid evolution towards resistance when additional mutations are selected under therapy [21]. In this study, the possible impact caused by statistically enriched NOPs found in CRF02_AG subtype was modelled within the context of a three-dimensional (3D) protein structure of the HIV-1CRF02-DNA-MG-DTG IN complex. Subsequently, stability predictions was performed using the mutation cut-off scanning matrix server (mCSM) to assess change in Gibbs free energy of mutations on the protein structure followed by interaction analysis to assesses the loss or gain of DTG interactions to Wild type and six mutated HIV-1CRF02-DNA_MG IN structures. Molecular modelling of HIV-1 CRF02 integrase sequences and DTG interaction analysis will help determine which mutations could affect the genetic barrier to the emergence of DTG drug resistance.

## Methods

### Generation of consensus HIV-1CRF02_AG Integrase sequence

To compare our study CRF02_AG treatment naïve sequences (*n* = 20) available in GenBank under the following accession numbers: MN816445- MN816488 [2], with INSTIs treatment naïve CRF02_AG IN sequences from Cameroon between 1994 and 2010. We performed a search on the HIV Los Alamos National Laboratory database (LANL) database for additional (*n* = 287) INSTI treatment Naïve patients’ sequences (https://www.hiv.lanl.gov/components/sequence/HIVsearch.com), completed on 01 February 2020. All Cameroonian HIV-1 subtype CRF02_AG IN sequences for treatment naïve patients, were included in our search criteria [14]. We selected one sequence per patient and every problematic sequence were excluded from further analyses. The consensus sequence was generated using the database-derived HIV-1 CRF02_AG sequences (*n* = 287) and CRF02_AG cohort sequences from our previous study (*n* = 20) [20]. An online quality control program that is available on the HIVLANL database (https://www.hiv.lanl.gov/content/sequence/QC/index.htm) was employed to screen nucleotide sequences for quality and to verify for stop codons, insertions and/or deletions. The MAFFT version 7 sequence alignment tool, was used to perform a multiple sequence alignment from which the consensus sequence was derived [22]. In an effort to eliminate possible contamination, which was part of our quality control measure, each of the viral sequences were inferred on a phylogenetic tree.

### HIV-1 subtyping using online programs

HIV-1 subtyping based on IN sequences was performed using two online available programs; REGA version 3 (http://www.bioafrica.net/subtypetool/html/subtypinghiv.html) and COMET-HIV (https://comet.lih.lu/).

### Drug resistance analysis

Mutations associated with resistance to INSTIs were identified using the Stanford University genotypic resistance interpretation algorithm, HIVdb version 8.3 (http://hivdb.stanford.edu/). All drug resistance mutations results were classified as either major or minor mutations, last accessed 01 April 2020.

### Homology modelling and quality assessment

The crystal structure of the HIV-1B intasome (nucleoprotein complex: containing viral DNA ends and the viral integrase protein) (PDBID: 5U1C) was used as a homologous template to generate a three-dimensional tetrameric structure of HIV-1CRF02_AG IN using the consensus sequence of recombinant subtype CRF02_AG sequence that we generated. The SWISSMODEL webserver was used for model construction by first constructing a pairwise sequence-structure alignment between HIV-1C wild-type (WT) amino acid sequence and template 5U1C [22]. The quality of the resulting model was assessed using SWISSMODEL quality assessment scores; the Qualitative model energy analysis (QMEAN) and Global model quality estimate (GMQE) scores. The QMEAN score is a composite scoring function assessing the major geometrical aspects of protein structures by comparing the predicted protein model to experimental structures of similar size, scores close to zero suggest high compatibility to experimental structures. The GMQE score estimates the quality of the predicted model using the properties from the target-template alignment and the template structure. Values are between 1 and 0, with higher values indicating more reliable models [23]. The Root mean square deviation (RMSD) analysis was done to compare the predicted model to the homologous template (PDBID: 5U1C) and to determine if there were any structural similarity between the two structures, lower RMSD values suggest very little deviation in terms of the backbone between the two structures. We also used publicly available algorithms located at the SAVES webserver (https://servicesn.mbi.ucla.edu/SAVES/) namely; ERRAT [24] VERIFY3D [25] and PROCHECK [26] to assess the quality of the predicted protein model. ERRAT statistically interrogated the nonbonded atomic interactions of the given target against the interactions of refined structures, with higher scores above 50 indicating higher quality in the protein model. VERIFY3D determines the compatibility of a structure (3D) to its own amino acid sequence (1D), higher values for VERIFY3D indicates high compatibility. PROCHECK on the other hand generates an Ramachandran plot that assesses the stereochemical parameters in a protein structure and if the percentage of phi and psi dihedral angles within the protein structure is more than 90% then the protein model has favourable residue conformations.

### Change in free energy predictions and interaction analysis

The predicted 3D structure of HIV-1 CRF02_AG IN was energy minimized using GROMACS software [27] and the resulting structure was used to introduce resistance associated and accessory mutations identified from the Stanford University HIVdb using PyMol mutagenesis wizard. The WT structure and a text file specifying single mutations was used as input to the program, mutation cut-off scanning matrix (mCSM). The change in energy after introduction of a mutation was calculated using mCSM. mCSM uses graph-based distance patterns of neighbouring residues and calculates a Delta-delta G-score for the impact of the mutation on the protein network and provides a phenotypic assessment by annotating a mutation as either being destabilizing (negative value) or stabilizing (positive value). The loss or gain of interactions between the WT and mutant neighbouring residues was calculated using Pymol find polar contacts option. To determine the effect of the mutation on DTG drug binding to the IN structure we energy minimized the WT CRF02-AG-DNA_MG_DTG and mutant complexes using GROMACS. Afterwards, we only selected the mutant structures that showed changes in the number of polar contacts with neighbouring residues and calculated DTG interactions with CRF02-DNA-MG complex IN structures using the PyMol find polar contacts option.

## Results

### HIV-1 subtyping

**Fig. 1:**
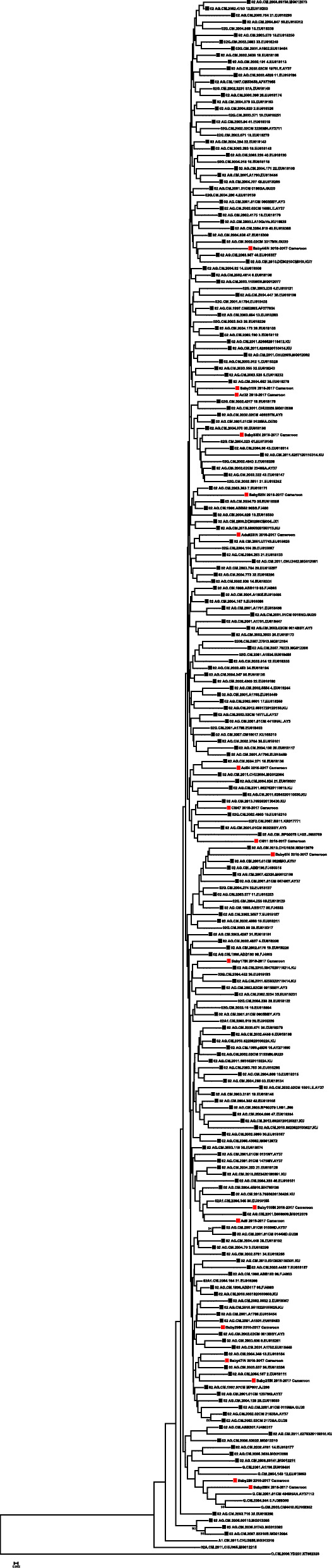
HIV-1 Integrase phylogenetic analysis inferred by ML. The Phylogenetic tree inferred in MEGA contains (*n* = 20) patient derived sequences in red box and (*n* = 287) online database sequence indicated in black box. HIV-1 reference sequences were acquired from the HIV-1 LANL database, using the 2010 data set. An ML tree was constructed using Mega version 7.0, with the Kimura 2 parameter. The alignment was based on HXB2 position 4351–5069, of approximately 700 bp in length. The percentage of replicate trees in which the associated taxa clustered together in the bootstrap test (1000 replicates) are shown next to the branches. The bootstrap values are above 70 indicating significant support for the branches simulated. The reference sequences are the unboxed sequences. All of the sequence’s clusters with HIV-1 subtype CRF02_AG. LANL, Los Alamos National Laboratory; MEGA, Molecular Evolutionary Genetics Analysis; ML, maximum likelihood

HIV-1 subtyping was done using HIV-1 subtyping online-automated tools and all sequences were verified using phylogenetic tree as HIV-1 subtype CRF02_AG (Fig. 1).

### Database derived IN sequence resistance analyses

After excluding multiple sequences from a patient to avoid overestimation of the variant calling and problematic sequences, we used 287 sequences collected between 1994 and 2010. These sequences were subsequently screened for the presence of RAMs. We identified 12.8% (37/287) sequences to contain RAMs, with only 1.0% (3/287) having major INSTI RAMs: T66A, Q148H, R263K and N155H. Two mutations, Q148H and R263K, occurred together in one sequence (0.3%), whereas T66A and N155H were present individually in one sequence each. A total of 11.8% (34/287) of the sequences contained five different IN accessory mutations, namely Q95K, T97A, G149A, E157Q and D232N. Mutations G149A and D232N occurred together in one sequence (0.3%). Notably, one sequence dating back from 2010 had two major mutations; Q148H and R263K in combination with two other minor mutations G149A and D232N.

### Generation of the consensus sequence for Cameroonian’s HIV-1 CRF02_AG subtype

**Fig. 2:**
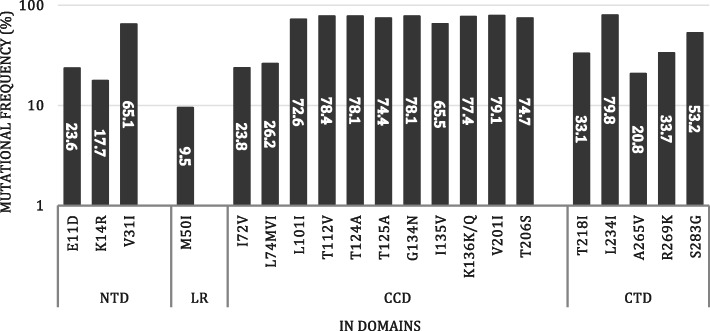
Prevalence of NOPs in IN genes from CRF02_AG subtypes. The figure shows the distribution of variants among the 287 and 20 CRF02_AG full length integrase sequences. Divided into: N-terminal domain (NTD) (residues 1–50), catalytic core domain (CCD) (residues 50–212) and C-terminal domain (CTD) composes of amino acids 213–288

The consensus sequences generated using the database-derived HIV-1 CRF02_AG sequences (*n* = 287) and cohort sequences (*n* = 20), identified 20 naturally occurring polymorphisms (NOPS): E11D,K14R, V31I, M50I, I72V, L74MVI, L101I, T112V, T124A, G134N, I135V, K136K/Q, V201I, T206S, T218I, L234I, A265V, R269K, S283G (Fig. 2). Three of these (E11D, K14R and V31I) belong to the NTD, whereas M50I belongs to the loop region connecting the NTD and CTD. Eleven NOPs (I72V, L74MVI, L101I, T112V, T124A, T124A, G134N, I135V, K136K/Q, V201I and T206S) are part of the CCD, and the remaining five (T218I, L234I, A265V, R269K and S283G) belong to the CTD.

### Molecular modelling and structural quality assessment

**Fig. 3:**
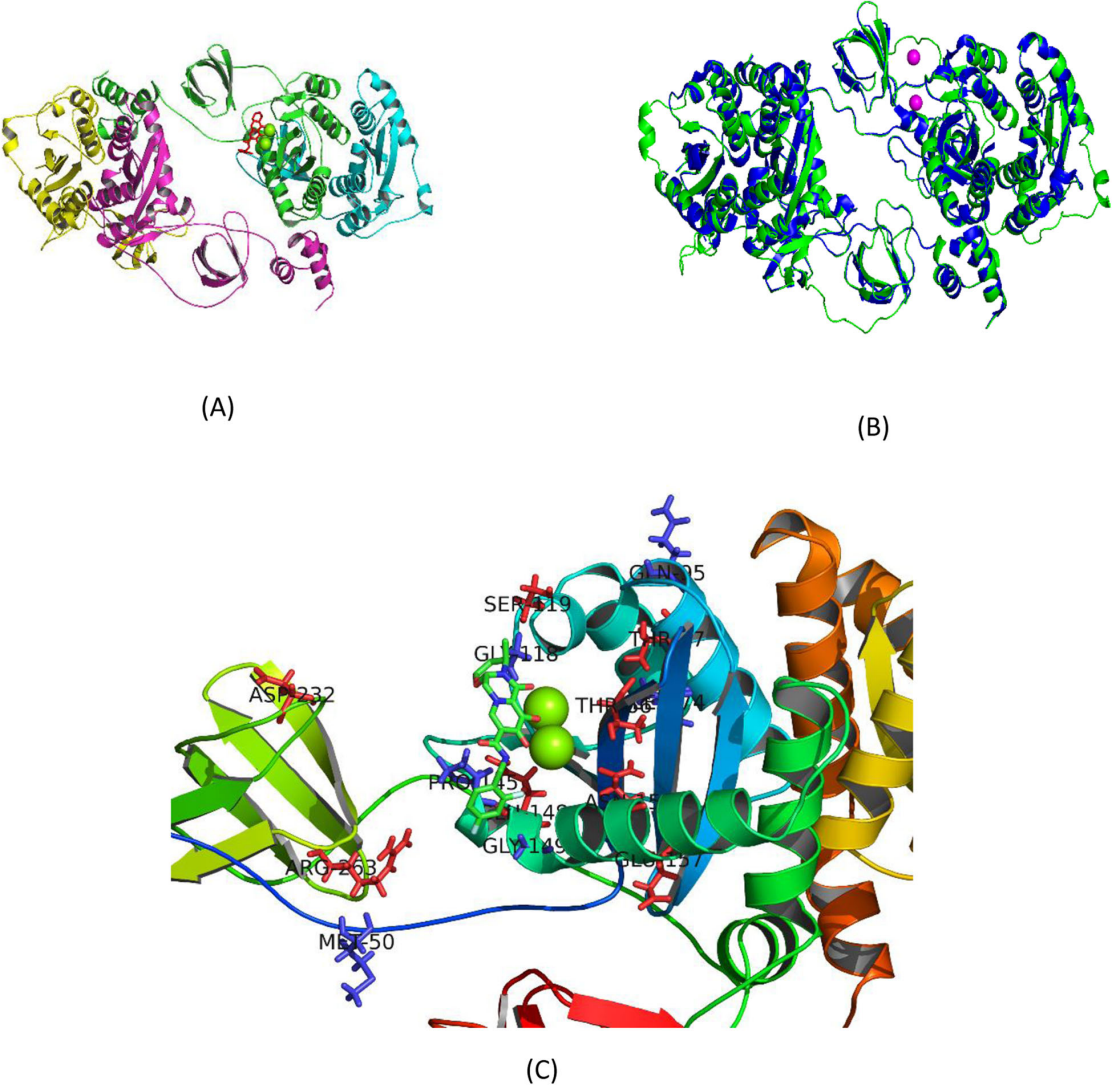
3D tetrameric structure for HIV-1 CRF02_AG IN. **a** Three-dimensional tetrameric structure predicted for HIV-1 CRF_02 AG IN in complex with magnesium ions. Chain A: green, Chain B: cyan, Chain C: Magenta, Chain D: yellow, Magnesium ions shown as spheres coloured in green and Dolutegravir shown as sticks coloured in red. No DNA shown. **b** Structural superimposition of HIV-1 AG IN onto HIV-1 B (5U1C) in complex with MG ions. CRF02_AG IN: green, 5U1C: blue, MG ions shown as magenta spheres. **c** Locations of stabilizing and destabilizing mutations on HIV-1 integrase CRF02_AG structure. Mutations that affect the protein structure are labelled and shown as red sticks and mutations with no effects are also labelled and shown as blue sticks. Magnesium ions shown as spheres coloured in green and Dolutegravir shown as sticks coloured by atom type

The sequence identity between the amino acid sequences of HIV-1 CRF02_AG sequence and the homologous template 5U1C was very high, approximately 93% and the sequence similarity was found to be 60% between the two sequences. The high sequence identity and coverage provides confidence in modelled regions of the protein structure and reduces the occurrence of any problematic or unresolved regions within the final protein model. Figure 3a, shows the 3D tetrameric structure for HIV-1 CRF02_AG IN that consist of 288 amino acids, 10 alpha helices, 9 beta sheets and 19 coil regions. The internal assessment scores calculated for the predicted model of HIV-1 CRF02_AG had an GMQE score of 0.10 and a QMEAN4 score of − 2.23, both scores confirming reliability of the modelled regions within the protein structure. Furthermore, the homology model passed most of the external 3D quality validation checks. The Verify3D score for the model was predicted to be 71.1% (acceptable for crude structures before energy minimization), while ERRAT score for all the chains was 86.0% and higher, the PROCHECK analysis indicated that 98.0% of residues occurred in most favoured and allowed regions of the Ramachandran plot, and the Prosa Z-score was − 6.18 which is in range with proteins of a similar size. Superimposing the template 5U1C onto the energy minimized structure of HIV-1 CRF02_AG indicated an RMSD value of 0.212 Å, suggesting very little backbone deviation in main chain atoms (Fig. 3b). Figure 3c, shows the locations of the 15 mutations relative to the active site.

### Gibbs free energy change and interaction analysis

**Fig. 4:**
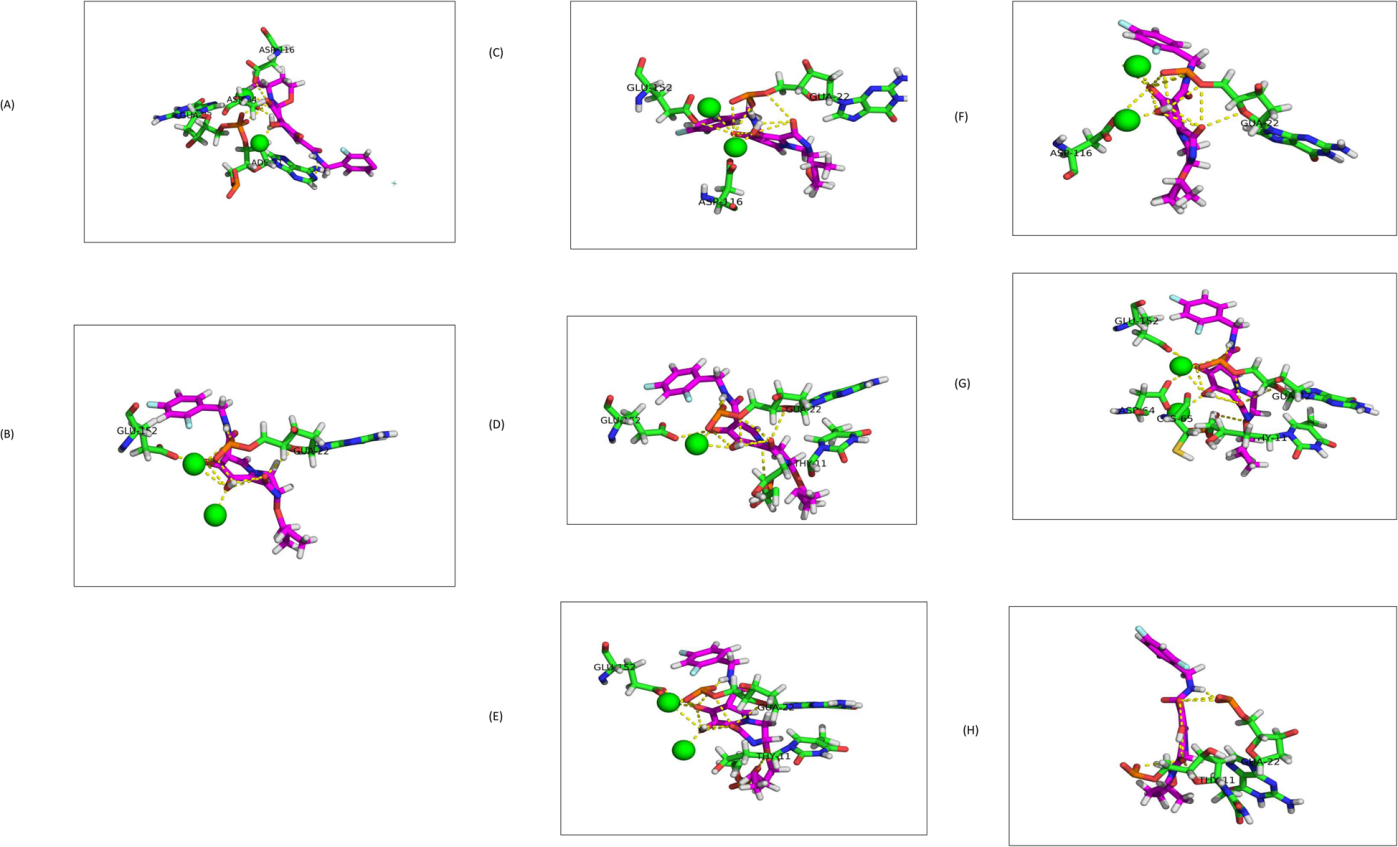
Interactions formed between DTG and the energy minimized WT and seven mutant structures for HIV-1 CRF02_AG Integrase. **a** WT HIV-1 CRF02_AG IN showing in total six contacts formed between DTG and two IN residues, two DNA nucleotides and two MG ions. **b** T66A HIV-1 CRF02_AG IN showing in total four contacts formed between DTG and one IN residue, one DNA nucleotide and two MG ions. **c** T97A HIV-1 CRF02_AG IN showing in total five contacts formed between DTG and two IN residues, one DNA nucleotide and two MG ions. **d** E157Q HIV-1 CRF02_AG IN showing in total four contacts formed between DTG and one IN residue, two DNA nucleotides and one MG ion. **e** Q148H HIV-1 CRF02_AG IN showing in total five contacts formed between DTG and one IN residue, two DNA nucleotides and two MG ions. **f** R263K HIV-1 CRF02_AG IN showing in total four contacts formed between DTG and one IN residue, one DNA nucleotide and two MG ions. **g** N155H HIV-1 CRF02_AG IN showing in total seven contacts formed between DTG and three IN residues, two DNA nucleotides and two MG ions. **h** D232N HIV-1 CRF02_AG IN showing in total two contacts formed between DTG and two DNA nucleotides. The drug DTG is shown as sticks and coloured in magenta, the MG ions are shown as spheres coloured in green while the IN protein residues and terminal 3’end viral DNA nucleotides are labelled and shown as sticks. Three letter codes for IN protein residues and numbers are given as well as the DNA nucleotide three letter codes and numbers. The nucleotides represent terminal end viral DNA nucleotides after 3′ processing

The mCSM predictions indicated that 14 of the 15 mutations (10 RAM’s and five accessory mutations), i.e. the M50I, T66A, L74I, L74M, T97A, G118S, S119R, P145S, Q148H, G149A, N155H, E157Q, D232N and R263K substitutions resulted in destabilizing effects of − 0.582, − 0.703 -1.069, − 0.93, − 1.051, − 0.492, − 0.091, − 0.485, − 0.133, − 0.421, − 0.975, − 1.111, − 0.512 and − 0.455 Kcal/Mol each, respectively. Only substitution Q95K resulted in a slightly stabilizing effect of 0.146Kcal/Mol. Interaction analysis of the single amino acid changes indicated differences in the number and type of interaction between neighbouring residues and the DNA. The T97A showed four polar contacts for T97 compared to the three of A97 (Table 1). This suggests a loss of stable contacts in this region that could destabilize the protein structure. Moreover, T66A, Q148H, N155H, D232N and R263K mutations all indicated a loss of interactions with neighbouring residues after the introduction of substitutions, while only the N155H mutation gained an additional interaction with the 3′ terminal viral DNA Adenine21 (Table 1). Inspection of the E157 residue showed four contacts with neighbouring residues while Q157 revealed five polar contacts of which two were with the 3′ terminal viral DNA (Table 1). In addition, the remaining other six substitutions; M50I, L74I, L74M, Q95K, G118S and P145S showed no changes in the number or type of interactions, implying no strong effect on the protein structure and function (Table 1). Protein drug interaction analysis of energy minimized complexes revealed interesting findings as accessory mutation E157Q made only one MG ion interaction and D232N none, while substitutions T66A, T97A, Q148H, R263K and N155H all had ionic interactions with two MG ions as well as with DDE motif active site residues and the 3′ terminal viral DNA nucleotides (Table 2) and (Figs. 4a-h). Most importantly is to note that MG ions are crucial for DTG coordination to displace viral DNA and thereby preventing HIV viral integration into host DNA.

**Table 1:** The number of polar contacts observed between WT residue and neighbouring residues before and after the introduction of the RAM’s and Accessory Mutations

**#**	**RAM’s**	**# Polar contacts**	
		**WT**	**Mutant**
1	M50I	None	None
2	T66A	2 (His67, Ile73)	1 (Ile73)
3	L74M	1 (Glu87)	1 (Glu87)
4	L74I	1 (Glu87)	1 (Glu87)
5	G118S	None	None
6	S119R	3 (Thy29, Asn120, Thr122)	3 (Thy29, **Glu92**, Thr122)
7	P145S	1 (Gln148)	1 (Gln148)
8	Q148H	3 (Pro145, Ser147, Val151)	1 (Pro145)
9	R263K	2 (Thy17, Cys56)	1 (Cys56)
10	N155H	4 (Val151, Glu152, Leu158, Lys159)	5 (Ade21, Val151, Glu152, Leu158, Lys159)
**#**	**Accessory Mutations**	**WT**	**Mutant**
1	Q95K	2 (Ala98, Tyr99)	2 (Ala98, Tyr99)
2	T97A	4 (Thr93, Gly94, I101)	3 (Thr93, Gly94, Ile101)
3	G149A	4 (Gua18, Gln146, Glu152, Ser153)	4 (Gua18, Gln146, Glu152, Ser153)
4	E157Q	4 (Ser153, Met154, Lys156, Ile161)	5 (**Thy20**, **Ade21**, Ser153, Met154, Ile161)
5	D232N	3 (Asp229, Ile234, Lys236)	2 (Asp229, Ile234)

The number in front of brackets is the total amount of interactions. Abbreviations used: *Ade* Adenine, *Ala (A)* Alanine, *Asp (D)* Aspartic acid, *Glu (E)* Glutamic acid, *Gly (G)* Glycine, *Gua* Guanine, *His (H)* Histidine, *Ile (I)* Isoleucine, *Leu (L)* Leucine, *Lys (K)* Lysine, *Met (M)* Methionine, *Asn (N)* Asparagine, *Gln (Q)* Glutamine, *Arg (R)* Arginine, *RAM’s* Resistance associated mutations, *Ser (S)* Serine, *Thr (T)* Threonine, *Thy* Thymidine, *Tyr (Y)* Tyrosine, *WT* Wild type. Bold indicates a change in amino acid and nucleotide. Three letter codes for IN protein residues and terminal end viral DNA nucleotides after 3′ processing are given

**Table 2:** Summary of all interactions observed between DTG and CRF_02AG IN for the WT and seven mutant structures

#	RAM’s/Accessory Mutations	CRF_02_AG IN	
		Hydrogen bonds	Ionic contact
1	WT	4 (Ade21, Gua22, Asp64, Asp116)	2 (MG)
2	T66A	2 (Gua22, Glu152)	2 (MG)
3	T97A	3 (Gua22, Asp116, Glu152)	2 (MG)
4	Q148H	3 (Thy11, Gua22, Glu152)	2 (MG)
5	N155H	5 (Thy11, Gua22, Asp64, Cys65, Glu152)	2 (MG)
6	**E157Q**	**3 (Thy11, Gua22, Glu152)**	**1 (MG)**
7	**D232N**	**2 (Thy11, Gua22)**	**None**
8	R263K	2 (Gua22, Asp116)	2 (MG)

Number outside bracket indicates total number of interactions. Abbreviations used: *Ade* Adenine, *Ala (A)* Alanine, *Asp (D)* Aspartic acid, *Cys (C)* Cysteine, *DTG* Dolutegravir, *Glu (E)* Glutamic acid, *Gly (G)* Glycine, *Gua* Guanine, *His (H)* Histidine, *Lys (K)* Lysine, *MG* Magnesium ions, *Asn (N)* Asparagine, *Gln (Q)* Glutamine, *Arg (R)* Arginine, *RAM’s* Resistance associated mutations, *Thr (T)* Threonine, *Thy* Thymidine, *WT* Wild type. In bold are the two accessory mutations that lost MG interactions crucial for DTG Binding. Three letter codes for IN protein residues and terminal end viral DNA nucleotides after 3′ processing are given

## Discussion

Despite INSTIs having an increased genetic barrier against resistance, studies performed from high-income countries shows that the occurrence of RAMS against INSTIs can happen, via acquired drug resistance mutations (DRM) and/or transmitted DRM, leading to reduced susceptibility to INSTIS and possible treatment failure [22, 28]. The IN mutations usually associated with reduced INSTIs susceptibility include both polymorphic mutations and non-polymorphic mutations [29, 30]. Other studies have reported that several NOPs can affect structural stability and flexibility of the IN protein structure [31, 32]. Previous researchers have reported low rates of IN mutations against INSTIs in Cameroon [15, 20]. The WHO has recommended the utilization of DTG as part of first-line regimens [16]. With the approval of INSTIs usage worldwide, it is predicted that approximately 57% of people living with HIV will be receiving DTG based regimens, including new-borns and children [33]. It is therefore imperative to screen for the presence of mutations against INSTIs which can affect treatment outcomes. Currently, there is limited data available for INSTI RAMs from studies that focuses on the SSA region, where over two-thirds of the presently infected individuals reside [34, 35]. In our previous studies we found low level of RAMSs against INSTIs [3].

In a recent study on CRF02_AG IN, we reported that accessory mutations can impact the binding of DTG with or without combination of primary resistance mutations [32, 36]. In this study we analysed the CRF02_AG IN gene sequences for the presence of polymorphic and non-polymorphic mutations. Four major INSTIs mutations were found within the database sequences: T66A, Q148H, N155H and R263K. R263K displayed moderate level resistance against EVG (12-fold) [37] and seems to confer low-level resistance against DTG. Structural analyses have suggested that DTG shares a similar interfacial mechanism of inhibition with EVG and RAL, but is able to make more intimate contacts with the viral DNA [38]. In addition, DTG can adapt its position and conformation in response to structural changes within the active site of EVG- or RAL resistant IN enzymes and in doing so avoid cross-resistance as a result of slower dissociation rates [39, 40]. Two principal mutation pathways identified from our study that reduces susceptibility to RAL are Q148H/K/R and N155H. These mutations are located in close proximity to the Integrase’s active site and each mutation significantly reduces viral fitness by 92-fold for Q148R, 30-fold for N155H [41]. Q148H and N155H mutations are thought to trigger conformational changes within the catalytic pocket that result in lower binding affinity of INSTIs to IN [42]. The variant T66A which is normally selected by EVG treatment, was detected in 0.3% of our sequence cohort. This variant is associated with 5-fold reduced susceptibility to EVG, however, T66A also bears cross-resistance to DTG and is selected by RAL [41]. Abraham et al., 2013, showed that the T66A mutant occurs within the two distal sheet from the DDE triad motif. The close proximity of the T66A/I/K variants to the viral DNA 3′ end and mutation N155H, could sterically hamper viral DNA binding and/or metal ion coordination with DTG [41]. The fact that only 1.0% of sequences analysed contained INSTI primary RAMs suggest that mutations against INSTIs will need to be monitored carefully against Cameroonians living with HIV. This result is in agreement with other studies done in Africa [20, 43–45] Asia [46, 47], Europe [48, 49] and South America [50] where studies showed a low frequency of INSTI primary RAMs.

In our study, we observed five IN accessory RAMs; namely Q95K, T97A, G149A, E157Q and D232N. T97A mutation can reduce EVG susceptibility by 3-fold [41] and combination of T97A mutation with other INSTI RAMs lead to reduced susceptibility to RAL [51, 52] and DTG [53, 54]. E157Q acts as a compensatory mutation and individually has a negligible effect on the susceptibility to INSTIs; however, a combination of E157Q with other INSTI RAMs may lead to reduced susceptibility to INSTIs [55, 56]. Individuals containing E157Q mutation in combination with other IN RAMs showed reduced susceptibility to DTG. Moreover, another rare nonpolymorphic accessory resistance mutation Q95K confers little if any effect on drug susceptibility to INSTIs [57]. A study by Axel Fun et al., 2010, showed that this secondary mutation enhances N155H-mediated resistance and partially restores the reduced replication caused by N155H [58]. In our study, we detected L74M mutations at a frequency > 20%, which is not surprising since, nearly 10% of ARV-Naïve patients infected with CRF02_AG viruses harbours L74M mutations [59]. This L74M mutation has minimal if any effect against susceptibly of INSTIs, but in combination with mutations at positions 140 and 148, it reduces susceptibility of DTG [38, 60, 61]. Within the IN CCD, we observed 11 of the reported INSTI NOPs. This IN region is important for recognition of DNA, binding and chromosomal integration of the newly synthesized double-stranded viral DNA into the host genomic DNA [62–64]. It contains the endonuclease and polynucleotide transferase site [62–64]. While in the CTD, a region that helps stabilize the integrase–viral DNA complex, five other NOP mutations were observed [65]. All of the afore mentioned mutations in either the CCD and/or CTD regions have the potential to affect the IN protein function and interfere with INSTIs binding [65].

We further analysed the effect of NOPs on the stability of the structures and neighbouring residues. Most of the variants noted in our study were shown to destabilise the protein structure, except for one mutation Q95K, that showed to exert a slightly stabilising effect on the protein structure and no changes in the number of polar contacts with neighbouring residues making it unlikely to affect the IN protein structure. It is known that destabilising effects of mutations on the protein structure might reduce drug binding. This was further explored by performing interaction analysis between the drug DTG and energy minimized structures of the WT and mutants T66A, T97A, Q148H, N155H, E157Q, R263K and D232N. The findings revealed accessory mutations E157Q and D232N had the potential to reduce and or prevent DTG binding to HIV-1 CRF02_AG IN structure based on the loss of MG ion interactions, while known RAM’s does not seem to influence DTG drug binding. However, the effect of RAM’s on DTG drug binding needs to be validated using molecular dynamic simulations to calculate the change in free energy of binding between DTG and HIV-1 CRF02_AG IN. Interestingly, the mutation E157Q occurred within the stable alpha-helix secondary structure element and made more contacts with DNA (stabilizing viral DNA complex), while the D232N mutation occurred within the stable Beta-sheet secondary structure element and in close proximity to the flexible G140’s loop region suggesting that these changes can affect the protein conformation and thereby interfere with drug binding leading to resistance.

A limitation of the study is the use of online database sequences that may contain contaminated and truncated sequences leading to spurious phylogenetic tree results and also these databases are not regularly updated. Furthermore, gaps in the aligned regions between the homologous template and target sequence may result in unresolved loop regions within the protein model which is one of the limitations of 3D protein modelling that can result in inaccurate interaction prediction. Furthermore, the interaction analysis was done for only a single static structure of the protein structure and does not consider the dynamic behaviour of the protein structure that might result in the loss and under estimation of crucial interaction partners. An important finding in this study is the fact that sequence diversity amongst different subtypes may affect different folding conformations of the HIV-1 IN subtypes thereby allowing not only RAM’s but accessory mutations to result in less efficacious INSTI binding to HIV-1 IN structures.

## Conclusion

Molecular modelling and interaction analysis provided novel insights into the effect of accessory mutations (E157Q and D232N) on HIV-1 CRF02_AG IN drug resistance. This emphasise the need to screen for the presence of INSTIs major RAM’s and accessory mutations in patients on INSTI treatment. This would help identify pathways that contribute to drug resistance and help tailor more effective treatment regimens in INSTI naïve patients.

## Data Availability

The datasets generated and/or analysed during the current study are available in the HIV Los Alamos National Laboratory database (LANL) (http://www.hiv.lanl.gov/) repository, and under NCBI database with GenBank accession numbers: MN816445- MN816488. The predicted 3D structural model of HIV-1 CRF02_AG IN is available from the corresponding author on reasonable request because we will use the predicted model in additional simulation studies.

## Abbreviations

IN: Integrase
INSTI: Integrase strand transfer inhibitors
NOP: Natural occurring polymorphism
SSA: Sub-Saharan Africa
RAMS: Resistance associated mutations
WHO: World Health Organisation
LANL: Los Alamos National Laboratory
CCD: Central core domain (CCD)
CTD: C-terminal domain
NTD: N-terminal domain
cART: combination antiretroviral therapy
PMCT: Prevention of mother-to-child transmission
FDA: Food and Drug Administration
RAL: Raltegravir
EVG: Elvitegravir
DTG: Dolutegravir
BIC: Bictegravir
3D: Three dimension
MRC: Medical Research Council
Mcsm: Mutation cut-off scanning matrix
